# Triglyceride-inflammation score established on account of random survival forest for predicting survival in patients with nasopharyngeal carcinoma: a retrospective study

**DOI:** 10.3389/fimmu.2024.1375931

**Published:** 2024-04-26

**Authors:** Jun Li, Yinxin Ye, Yonglin Cai, Huojin Ji, Weiling Qin, Yonglin Luo, Xiaoying Zhou, Zhe Zhang, Xue Xiao, Bin Zhang

**Affiliations:** ^1^Department of Clinical Laboratory, Wuzhou Red Cross Hospital, Wuzhou, Guangxi, China; ^2^Guangxi Health Commission Key Laboratory of Molecular Epidemiology of Nasopharyngeal Carcinoma, Wuzhou Red Cross Hospital, Wuzhou, Guangxi, China; ^3^Key Laboratory of High-Incidence-Tumor Prevention & Treatment (Guangxi Medical University), Ministry of Education, Nanning, Guangxi, China; ^4^Department of Otolaryngology-Head and Neck Surgery, First Affiliated Hospital of Guangxi Medical University, Nanning, Guangxi, China; ^5^Department of Radiation Oncology, Wuzhou Red Cross Hospital, Wuzhou, Guangxi, China

**Keywords:** nasopharyngeal carcinoma, lipid, triglyceride, inflammation, prognosis, nomogram

## Abstract

**Objective:**

This study aimed to establish an effective prognostic model based on triglyceride and inflammatory markers, including neutrophil-to-lymphocyte ratio (NLR), lymphocyte-to-monocyte ratio (LMR), and platelet-to-lymphocyte ratio (PLR), to predict overall survival (OS) in patients with nasopharyngeal carcinoma (NPC). Additionally, we aimed to explore the interaction and mediation between these biomarkers in their association with OS.

**Methods:**

A retrospective review was conducted on 259 NPC patients who had blood lipid markers, including triglyceride and total cholesterol, as well as parameters of peripheral blood cells measured before treatment. These patients were followed up for over 5 years, and randomly divided into a training set (n=155) and a validation set (n=104). The triglyceride-inflammation (TI) score was developed using the random survival forest (RSF) algorithm. Subsequently, a nomogram was created. The performance of the prognostic model was measured by the concordance index (C-index), time-dependent receiver operating characteristic (ROC) curve, and decision curve analysis (DCA). The interaction and mediation between the biomarkers were further analyzed. Bioinformatics analysis based on the GEO dataset was used to investigate the association between triglyceride metabolism and immune cell infiltration.

**Results:**

The C-index of the TI score was 0.806 in the training set, 0.759 in the validation set, and 0.808 in the entire set. The area under the curve of time-dependent ROC of TI score in predicting survival at 1, 3, and 5 years were 0.741, 0.847, and 0.871 respectively in the training set, and 0.811, 0.837, and 0.758 in the validation set, then 0.771, 0.848, and 0.862 in the entire set, suggesting that TI score had excellent performance in predicting OS in NPC patients. Patients with stage T1-T2 or M0 had significantly lower TI scores, NLR, and PLR, and higher LMR compared to those with stage T3-T3 or M1, respectively. The nomogram, which integrated age, sex, clinical stage, and TI score, demonstrated good clinical usefulness and predictive ability, as evaluated by the DCA. Significant interactions were found between triglyceride and NLR and platelet, but triglyceride did not exhibit any medicating effects in the inflammatory markers. Additionally, NPC tissues with active triglyceride synthesis exhibited high immune cell infiltration.

**Conclusion:**

The TI score based on RSF represents a potential prognostic factor for NPC patients, offering convenience and economic advantages. The interaction between triglyceride and NLR may be attributed to the effect of triglyceride metabolism on immune response.

## 1 Introduction

Nasopharyngeal carcinoma (NPC) is a malignant head and neck tumor that has a high incidence in southern China and Southeast Asia (1). There is a double or triple incidence of NPC among males compared to females in most populations (1, 2). According to a comprehensive assessment of the global cancer burden in 2022 (3) conducted by CANCER TODAY of the International Agency for Research on Cancer, a branch of the World Health Organization, the worldwide age-standardized rates of NPC were 1.9 per 100 000 person-years for males and 0.73 per 100 000 person-years for females. In Asia, the age-standardized rates of NPC were higher, with 2.6 per 100 000 person-years for males and 0.98 per 100 000 person-years for females. Radiation therapy is sensitive to NPC. Due to individual heterogeneity, patients with the same TNM stage may have different outcomes. The identification of serum tumor markers could aid in the noninvasive prediction of prognosis for NPC.

Reprogramming cellular metabolism is a key feature of the rapid proliferation of cancer cells (4), and the deregulation of lipid metabolism is one of the most common metabolic changes. We analyzed the lipid spectrum in the plasma of the subjects using liquid chromatography-tandem mass spectrometry (LC-MS/MS) and found significant differences in the lipid profiles between NPC patients and healthy controls (5). Previous studies have reported that pretreatment serum low-density lipoprotein cholesterol (LDL-C) and high-density lipoprotein cholesterol (HDL-C) are negative and favorable independent prognostic factors for NPC, respectively (6, 7). Chen et al. utilized widely targeted lipidomics to measure the plasma lipid profiles of patients with locoregionally advanced NPC. They identified six plasma lipid predictors for survival, which were significantly associated with inflammation-related markers such as C-reactive protein and white blood cell (WBC) count (8).

Tumor-promoting inflammation is also an enabling characteristic of cancer. Studies have reported that the inflammatory markers including neutrophil-to-lymphocyte ratio (NLR), lymphocyte-to-monocyte ratio (LMR), and platelet-to-lymphocyte ratio (PLR) can reflect the body’s inflammatory status and have prognostic value in various cancers (9–12). In addition, it has been observed that a lower PLR is significantly associated with a higher pathological response rate for neoadjuvant combination therapy based on tislelizumab for muscle-invasive bladder cancer (13). Our previous research showed that high NLR, high PLR, and low LMR were significantly associated with poorer overall survival (OS) of NPC (14), similar to the studies conducted by Chen Y et al. (15) and Xu F et al. (16).

Patients with NPC should undergo routine blood tests to assess anemia, infection, and immune function, as well as blood lipid detection to address abnormal lipid levels promptly, mitigate cachexia, and prevent cardiovascular complications. It is easy to obtain through routine blood tests for lipid markers and the parameters of peripheral blood cell measurements in clinical practice. The integration of lipid markers and peripheral blood cell parameters allows for a comprehensive evaluation of prognostic value in NPC. However, most lipid or inflammatory markers were assessed separately, and the prognostic model was built using univariate and multivariate analyses, which had limitations in handling the collinearity among variables. The random survival forest (RSF) method, as the emerging machine learning algorithm, is not sensitive to multicollinearity and can significantly improve prediction performance. In this study, we aimed to construct a lipid-inflammation scoring model based on the RSF algorithm and estimate its performance in refining patient stratification, then compare it with the nutritional scores reported by others, such as controlling nutritional status (CONUT) score and prognostic nutritional index (PNI). Subsequently, a nomogram was constructed to predict the prognosis of NPC efficiently and conveniently. Additionally, the interaction and mediation between the biomarkers in their association with OS were analyzed.

## 2 Methods

### 2.1 Patients enrolled

The patient’s data were collected retrospectively from February 2009 to December 2017 at Wuzhou Red Cross Hospital, China. The inclusion criteria for this study were as follows: (1) patients newly diagnosed with primary NPC based on pathological findings, (2) no prior anticancer treatment, and (3) fasting lipids test and routine blood test conducted before treatment. The exclusion criteria were: (1) prior malignancy, (2) metabolic diseases, and (3) infection or inflammation. The cancer TNM stage was determined according to the Chinese 2008 staging system (**Supplementary Table 1**). Patients with stages I-II received radiotherapy alone, while those with stages III-IV received radiotherapy in combination with chemotherapy. This study was approved by the Ethics Committee of Wuzhou Red Cross Hospital (NO. LL2021-53) and informed consent was waived due to its retrospective design.

**Figure 1:**
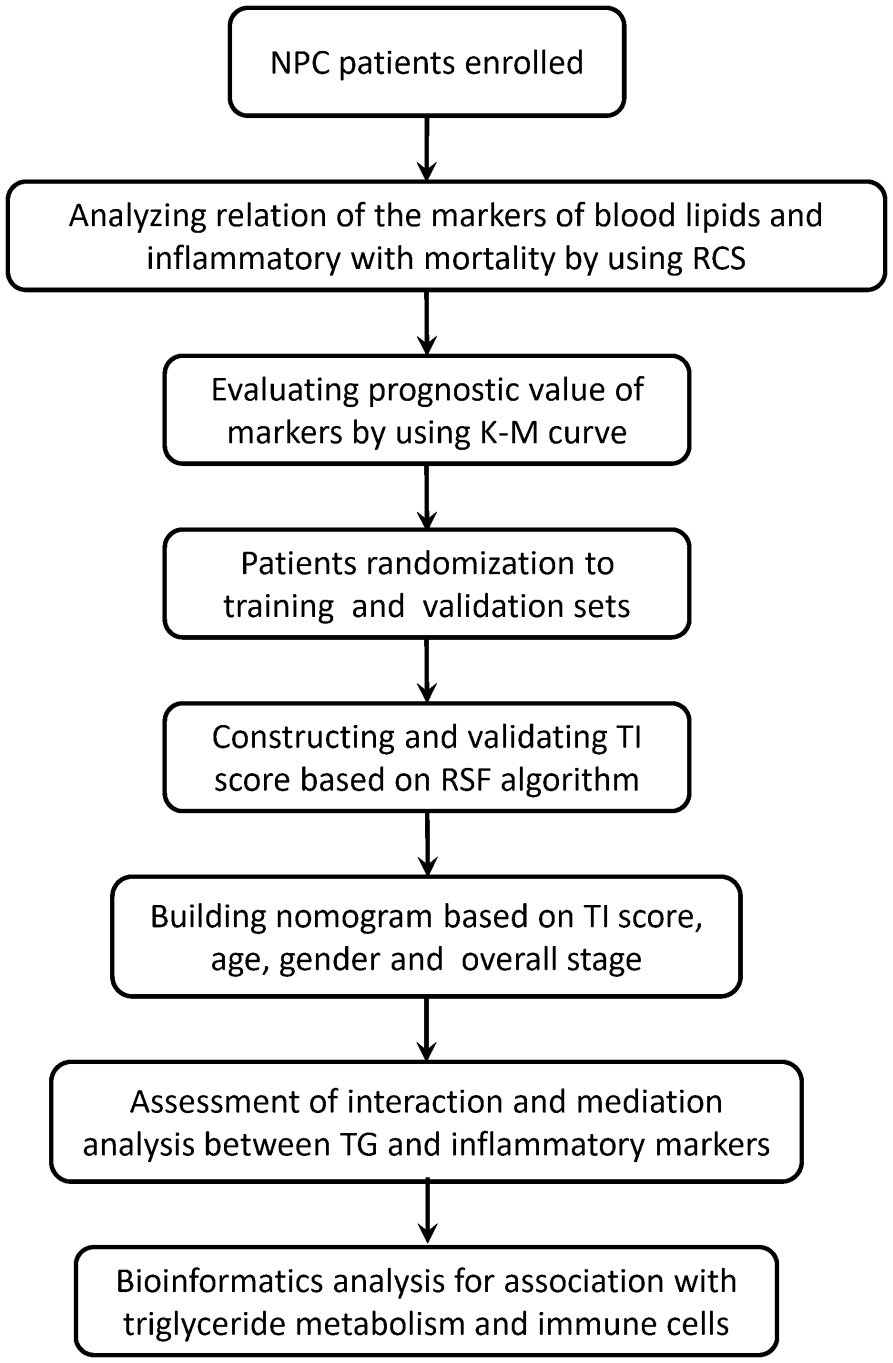
The workflow chart of this study. RCS, restricted cubic splines; RSF, random survival forest; TI, Triglyceride-inflammation.

The workflow chart of this study is shown in **Figure 1**. The NPC patients were randomly assigned to a training set and a validation set. The triglyceride-inflammation (TI) score based on the RSF algorithm was developed in the training set and validated in the validation set. The performance of the TI score was measured by the concordance index (C-index), time-dependent receiver operating characteristic (ROC) curve, and decision curve analysis (DCA).

### 2.2 Blood tests

Fasting blood samples were collected from each patient before initiating treatment. The blood lipid markers, including triglyceride (TG), total cholesterol (CHOL), high-density lipoprotein-cholesterol (HDL-C), and low-density lipoprotein-cholesterol (LDL-C) and serum albumin were measured using a MODULAR DP automatic biochemical analyzer (Roche, Germany). The parameters of peripheral blood cells, including WBC, lymphocytes, neutrophils, monocytes, and platelets, were detected using an XE-2100 automatic hematology analyzer (Sysmex, Japan). All assays were performed according to the instruction manual of the reagent kits.

### 2.3 Follow-up

The OS was defined as the time from diagnosis to either death or the end of follow-up (December 31, 2021).

### 2.4 Bioinformatics analysis of triglyceride metabolism

The GSE102349 dataset, downloaded from the Gene Expression Omnibus (GEO) website, is consists of 113 NPC tissues and prognostic data for 88 patients with progression-free survival (PFS) (17). The geneset for triglyceride metabolism included 2 key synthesis genes (AGPAT1 and DGAT1) and 3 key degradation genes (ATGL, HSL, and MGL). We performed a consensus clustering analysis on the geneset of triglyceride metabolism in the GSE102349 dataset and identified distinct patterns of triglyceride metabolism. We analyzed the differences in parameters of NPC patients with different triglyceride metabolism subtypes, including PFS, the immune microenvironment infiltration signature [based on EPIC (Estimate the Proportion of Immune and Cancer cells) (18) or MCPcounter (Microenvironment Cell Populations counter) (19)], and the immune score (calculated by ESTIMATE (20) or xCell (21) algorithms).

### 2.5 Statistical analysis

The statistical analyses and visualization of the data were performed using R version 4.3.0 software from the R Foundation for Statistical Computing. To calculate the NLR, the neutrophil count was divided by the lymphocyte count. The LMR was calculated by dividing the lymphocyte count by the monocyte count. The PLR was calculated by dividing the platelet count by the lymphocyte count. The CONUT score (22) was calculated by the formula: serum albumin score + total lymphocyte count score + total cholesterol score (**Supplementary Table 2**). The PNI (23) was calculated by the formula: albumin (g/L) + 5 × lymphocyte count × 10^9^/L. The markers were presented as median (interquartile range), and differences between the two groups were compared using the Wilcoxon test. To assess the dose-response relationship between the markers and mortality in NPC patients, a restricted cubic splines (RCS) curve was applied using the R package “rms”. Lipid-inflammation score was constructed using the RSF algorithm with the R package “randomForestSRC” (24). The prediction abilities of the lipid-inflammation score were assessed using the C-index and the area under the curve (AUC) of the time-dependent ROC curve with the R package “timeROC” (25). The optimal cutoff values of the markers were determined using the R package “survminer”. The OS of NPC patients between different groups was calculated using the Kaplan-Meier survival curve with the R packages “survminer” and “survival” (26). The hazard ratio (HR) and 95% confidence interval (CI) were estimated. A nomogram was constructed using the R package “rms”, and its prognostic efficiency was evaluated using DCA with the R package “ggDCA”. The interaction between inflammatory markers and triglyceride, as well as the mediation analysis, were performed using the R packages “visreg” (27) and “mediation” (28), respectively. The Consensus Clustering analysis was conducted using the R package “ConsensusClusterPlus” (29). The assessment of immune cell infiltration of NPC tissues was performed using the R package “IOBR” (30). A two-sided p-value < 0.05 was considered statistically significant.

## 3 Results

### 3.1 Clinical characteristics of patients

A total of 259 patients with NPC were enrolled and their characteristics were described in **Table 1**. The age of patients ranged from 28 to 84 years. The age and sex distribution of these enrolled patients fit the population characteristics in high-incidence areas (1). The majority of cases (95.4%, 247/259) were non-keratinizing carcinoma. The median follow-up period was 60.0 months, during which 94 cases died. The 1-year, 3-year, and 5-year OS rates were 92.3% (95% CI: 89.1%-95.6%), 75.7% (95% CI: 70.6%-81.1%), and 68.7% (95% CI: 63.2%-74.6%), respectively.

**Table 1:** Baseline characteristics of patients with NPC [n (%) or median (interquartile range)].

Characteristics	Patients			P value
	Total (n=259)	Training set (n=155)	Validation set (n=104)	
Age	52.0 (44.0, 59.5)	52.0 (44.0, 59.0)	50.5 (43.8, 60.0)	0.886
Sex				0.507
Female	68 (26.3%)	43 (27.7%)	25 (24.0%)	
Male	191 (73.7%)	112 (72.3%)	79 (76.0%)	
T stage				0.956
T1	37 (15.1%)	22 (15.1%)	15 (15.2%)	
T2	63 (25.7%)	37 (25.3%)	26 (26.3%)	
T3	69 (28.2%)	43 (29.5%)	26 (26.3%)	
T4	76 (31.0%)	44 (30.1%)	32 (32.3%)	
Missing	14	9	5	
N stage				0.325
N0	18 (7.3%)	11 (7.5%)	7 (7.1%)	
N1	40 (16.3%)	29 (19.9%)	11 (11.1%)	
N2	149 (60.8%)	84 (57.5%)	65 (65.7%)	
N3	38 (15.5%)	22 (15.1%)	16 (16.2%)	
Missing	14	9	5	
M stage				0.74
M0	229 (93.5%)	135 (92.5%)	94 (94.9%)	
M1	13 (5.3%)	9 (6.2%)	4 (4.0%)	
Mx	3 (1.2%)	2 (1.4%)	1 (1.0%)	
Missing	14	9	5	
Overall stage				0.731
I	8 (3.3%)	6 (4.1%)	2 (2.0%)	
II	18 (7.3%)	12 (8.2%)	6 (6.1%)	
III	114 (46.5%)	67 (45.9%)	47 (47.5%)	
IV	105 (42.9%)	61 (41.8%)	44 (44.4%)	
Missing	14	9	5	
Status				0.844
Alive	165 (63.7%)	98 (63.2%)	67 (64.4%)	
Dead	94 (36.3%)	57 (36.8%)	37 (35.6%)	
Triglyceride (mmol/L)	1.24 (0.93, 1.84)	1.26 (0.91, 1.92)	1.23 (0.96, 1.82)	0.722
Female	1.00 (0.80, 1.38)	0.93 (0.74, 1.36)	1.03 (0.92, 1.51)	0.152
Male	1.30 (0.97, 2.07)	1.37 (0.97, 2.12)	1.26 (0.98, 2.02)	0.699
CHOL (mmol/L)	5.07 (4.25, 5.67)	5.11 (4.32, 5.67)	4.95 (4.16, 5.65)	0.322
Female	4.59 (3.96, 5.56)	4.57 (4.00, 5.57)	4.82 (3.89, 5.50)	1.000
Male	5.11 (4.40, 5.70)	5.19 (4.51, 5.74)	4.95 (4.28, 5.65)	0.136
HDL-C (mmol/L)	1.22 (1.05, 1.45)	1.26 (1.09, 1.50)	1.20 (1.05, 1.40)	0.174
Female	1.33 (1.18, 1.50)	1.38 (1.18, 1.59)	1.29 (1.17, 1.45)	0.652
Male	1.19 (1.00, 1.39)	1.23 (1.02, 1.46)	1.15 (1.00, 1.38)	0.278
LDL-C (mmol/L)	3.31 (2.59, 3.84)	3.40 (2.66, 3.86)	3.25 (2.57, 3.76)	0.336
Female	2.94 (2.20, 3.74)	2.76 (2.31, 3.68)	3.10 (2.18, 3.77)	0.656
Male	3.40 (2.73, 3.95)	3.47 (2.81, 4.14)	3.25 (2.62, 3.74)	0.097
WBC (10^9^/L)	6.91 (5.60, 8.52)	6.80 (5.55, 8.80)	7.10 (5.64, 8.14)	0.772
Female	6.75 (4.87, 8.00)	6.70 (4.80, 8.06)	6.80 (5.26, 7.80)	0.919
Male	7.00 (5.92, 8.75)	6.85 (6.08, 8.91)	7.10 (5.85, 8.31)	0.725
NLR	2.48 (1.83, 3.42)	2.48 (1.78, 3.59)	2.49 (1.85, 3.31)	0.818
Female	2.23 (1.72, 3.57)	2.22 (1.55, 3.59)	2.25 (2.11, 3.05)	0.471
Male	2.51 (1.88, 3.41)	2.50 (1.94, 3.58)	2.53 (1.81, 3.33)	0.969
LMR	3.83 (2.98, 5.12)	3.84 (3.15, 5.21)	3.81 (2.95, 4.83)	0.545
Female	4.29 (3.27, 5.89)	4.34 (3.33, 6.15)	4.00 (3.09, 5.35)	0.558
Male	3.70 (2.95, 4.88)	3.69 (3.02, 5.11)	3.74 (2.94, 4.68)	0.79
PLR	148 (109, 188)	150 (114, 187)	142 (107, 188)	0.404
Female	160 (140, 223)	159 (138, 225)	168 (142, 206)	0.944
Male	136 (107, 182)	145 (109, 184)	130 (106, 177)	0.357

NPC, nasopharyngeal carcinoma; SD, standard deviation; LDL-C, low-density lipoprotein cholesterol; HDL-C, high-density lipoprotein cholesterol; WBC, white blood cell; NLR, neutrophil to lymphocyte ratio; LMR, lymphocyte to monocyte ratio; PLR, platelet to lymphocyte ratio.

These patients were randomly divided into a training set and a validation set at a ratio of 6:4 by using the sample function of the R package “base”. There were no significant differences in the clinicopathologic parameters between the training set and validation set (**Table 1**).

### 3.2 High triglyceride, NLR, PLR, and low LMR are significantly associated with poor outcomes in NPC patients

**Figure 2:**
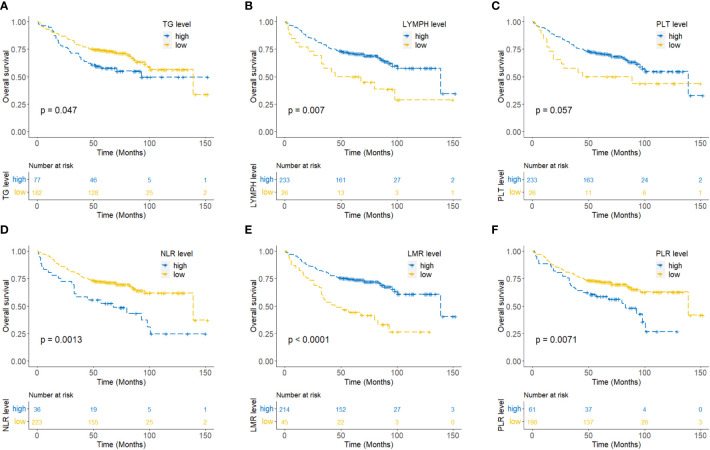
Kaplan-Meier curves for overall survival of NPC patients according to triglyceride **(A)**, lymphocyte count **(B)**, platelet count **(C)**, neutrophil to lymphocyte ratio (NLR) **(D)**, lymphocyte to monocyte ratio (LMR) **(E)**, and platelet to lymphocyte ratio (PLR) **(F)**.

To explore the association between circulating lipids, inflammatory markers, and the outcomes of NPC patients, we first utilized the RCS curve to model and visualize the relationship between lipids (triglyceride, cholesterol, HDL-C, and LDL-C), inflammatory markers (lymphocytes, neutrophils, monocytes, platelets, NLR, LMR, and PLR) with mortality in the entire set (**Supplementary Figure 1**). Our data demonstrated a gradual increase in the mortality risk of NPC patients with increasing levels of triglyceride, NLR, and PLR, while a gradual decrease in the mortality risk was observed with increasing lymphocyte count, platelet count, and LMR (p<0.05). There was no significant correlation between mortality and the markers of cholesterol, HDL-C, LDL-C, neutrophil count, and monocyte count (p>0.05). Additionally, Kaplan-Meier survival curve analysis revealed that NPC patients tend to have significantly poorer OS when exhibiting with higher levels of triglyceride, NLR, and PLR or lower levels of lymphocyte count and LMR (p<0.05) (**Figure 2**).

### 3.3 Triglyceride-inflammation score is a novel prognosticator for OS of NPC patients

**Figure 3:**
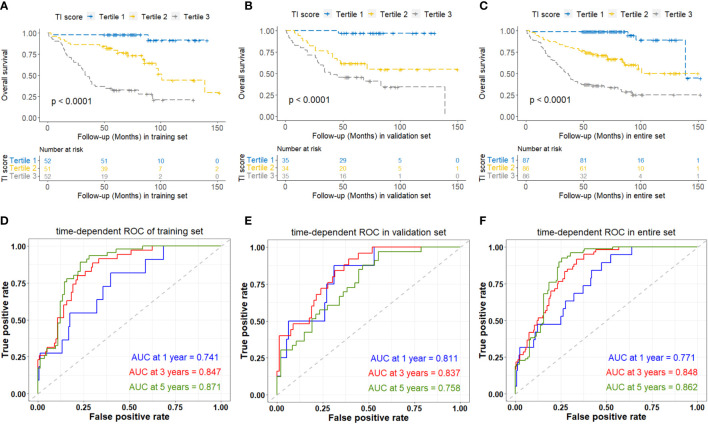
Kaplan-Meier and time-dependent ROC curves analyses based on TI score of overall survival of NPC patients in the training set **(A, D)**, validation set **(B, E)**, and entire set **(C, F)**. TI, Triglyceride-inflammation.

Considering that the lymphocyte count is reflected by parameters NLR, LMR, and PLR, we utilized the RSF algorithm to construct the TI score in the training set, based on the level of triglyceride, NLR, LMR, and PLR which showed significant prognostic value for each NPC patient (**Supplementary Figure 2**). After parameter tuning, we found that when the ntree was set to 1000, the error rate of the model tended to be stable. The C-index was 0.806 (95%CI: 0.753-0.860) in the training set, 0.759 (95%CI: 0.688-0.829) in the validation set, and 0.808 (95%CI: 0.768-0.848) in the entire set. Patients were then classified into low, medium, and high TI score groups based on the tertile of TI score. Kaplan-Meier curve analysis for all the training set, validation set, and entire set indicated that there was a significant difference in OS among the low (tertile 1), medium (tertile 2), and high (tertile 3) TI score groups (**Figures 3A-C**), suggesting a negative association between survival time and TI score. The time-dependent ROC analyses were performed to evaluate the accuracy of the TI score in predicting survival at 1, 3, and 5 years, and the AUC were 0.741, 0.847, and 0.871 respectively in the training set, and 0.811, 0.837, and 0.758 in the validation set, then 0.771, 0.848, and 0.862 in the entire set (**Figures 3D-F**). These results demonstrated that the TI score performed well in predicting survival with good sensitivity and specificity. In the entire set, the patients with stage T1-T2 or M0 exhibited significantly lower TI score, NLR, and PLR, as well as higher LMR compared to those with stage T3-T4 or M1, respectively (p < 0.05) (**Table 2**).

**Table 2:** Association of pretreatment NLR, LMR, PLR, and TI score with clinicopathologic characteristics in the entire set of NPC patients [median (interquartile range)].

Characteristic	n	Triglyceride (mmol/L)	NLR	LMR	PLR	TI score
Age (year)						
<52	129	1.29 (0.95, 1.91)	2.36 (1.71, 3.42)	3.85 (3.09, 5.35)	142.47 (110.92, 187.86)	12.12 (9.30, 17.76)
≥52	130	1.14 (0.91, 1.66)	2.58 (2.02, 3.40)	3.74 (2.90, 4.81)	149.75 (107.50, 187.51)	13.14 (10.42, 17.94)
Sex						
Female	68	1.00 (0.80, 1.38)	2.23 (1.72, 3.57)	4.29* (3.27, 5.89)	159.80*** (140.03, 222.94)	12.09 (9.31, 15.50)
Male	191	1.30 (0.97, 2.07)	2.51 (1.88, 3.41)	3.70 (2.95, 4.88)	135.86 (106.78, 182.34)	12.89 (9.80, 18.26)
T stage						
T1-T2	100	1.29 (0.94, 1.99)	2.25* (1.71, 3.20)	4.00** (3.33, 5.70)	140.36* (102.98, 171.70)	11.76* (9.01, 15.51)
T3-T4	145	1.22 (0.93, 1.72)	2.58 (1.86, 3.39)	3.65 (2.90, 4.82)	150.23 (115.56, 192.37)	13.08 (10.58, 18.78)
N stage						
N0-N1	58	1.09 (0.83, 1.67)	2.33 (1.85, 3.02)	4.00 (3.34, 6.08)	151.74 (120.18, 182.43)	11.98 (9.44, 16.48)
N2-N3	187	1.26 (0.95, 1.86)	2.50 (1.78, 3.41)	3.80 (2.98, 5.08)	142.47 (105.89, 187.86)	12.85 (9.31, 17.53)
M stage						
M0	229	1.22 (0.93, 1.81)	2.35** (1.75, 3.25)	3.93*** (3.09, 5.35)	145.09* (107.39, 183.11)	12.11*** (9.30, 16.31)
M1	13	1.40 (1.17, 1.83)	4.38 (2.58, 6.23)	2.51 (1.51, 3.32)	183.44 (135.86, 257.80)	24.58 (13.32, 30.23)
Mx	3					
Overall stage						
I-II	26	1.04 (0.81, 1.73)	2.20 (1.85, 2.49)	4.18* (3.47, 6.33)	130.36 (107.50, 160.56)	11.25 (8.85, 14.02)
III-IV	219	1.26 (0.95, 1.84)	2.51 (1.80, 3.42)	3.80 (2.96, 5.08)	147.52 (108.28, 189.59)	12.58 (9.44, 18.01)

NPC, nasopharyngeal carcinoma; NLR, neutrophil to lymphocyte ratio; LMR, lymphocyte to monocyte ratio; PLR, platelet to lymphocyte ratio; RSF, random survival forests; TI, Triglyceride-inflammation; *p < 0.05; **p < 0.01; ***p < 0.001.

Except for PLR, no differences were found in the levels of triglyceride, NLR, LMR, or TI score between male and female patients (**Table 2**). And stratified survival analysis by sex in the entire set (**Supplementary Figure 3**) showed that the C-index of TI score in the male and female subgroups were 0.780 (95%CI: 0.732-0.829) and 0.790 (95%CI: 0.697-0.882), respectively, without significant difference. These results indicated that the TI score might not be influenced by sex.

Among 259 NPC patients, 229 patients were detected for serum albumin before treatment. There were no significant differences in the clinicopathologic parameters between the albumin subset and the entire set (**Supplementary Table 3**). The survival analysis for the albumin subset showed that the C-index of CONUT score and PNI were 0.576 (95%CI: 0.513-0.640) and 0.591 (95%CI: 0.523-0.659), respectively; and the AUC in predicting survival at 1, 3, and 5 years were 0.693, 0.606 and 0.561 for CONUT score, and 0.717, 0.628 and 0.565 for PNI (**Supplementary Figure 4**). The prognostic efficacy of the CONUT score and PNI were worse than that of the TI score.

### 3.4 Nomogram based on TI score predicts the survival of NPC patients effectively

**Figure 4:**
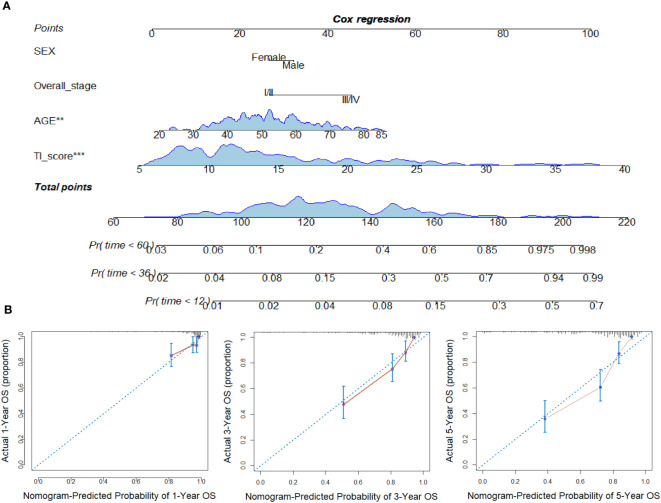
Construction of nomogram in NPC. **(A)** Nomogram for predicting 12, 36, and 60 months of overall survival in NPC patients. **(B)** Calibration curves for predicting the fitness of the nomogram in 12, 36, and 60 months. **p < 0.01; ***p < 0.001.

A COX regression model was further developed based on age, sex, overall stage, and TI score as variables. The resulting COX survival model was then used to create a nomogram, which was displayed in **Figure 4A**. Calibration curves were generated to assess the accuracy of the model in predicting the 1-, 3- and 5-year OS and these curves demonstrated good discrimination (**Figure 4B**). To provide an unbiased estimate of model performance, we conducted an internal validation using a bootstrap resampling process with B=1000 (using the “validate” function of R package “rms”). The C-index and corrected C-index were calculated as 0.795 and 0.784, respectively.

**Figure 5:**
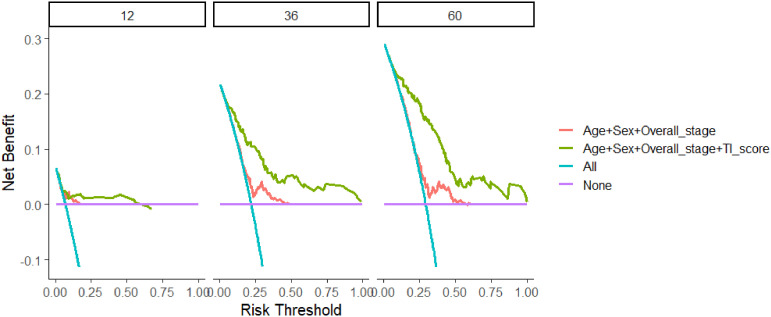
Comparison of decision curve analysis between the model based on age, sex, and overall stage and TI score-based model in 12, 36, and 60 months. TI, Triglyceride-inflammation.

DCA was conducted to compare the predictive performance of the model incorporating age, sex, overall stage, and TI score (**Figure 5**). The net benefits of the prognostic model at 12, 36, and 60 months were found to be superior to those of the traditional model.

### 3.5 Statistical interaction but not mediation between triglyceride and NLR in the association with OS of NPC

**Figure 6:**
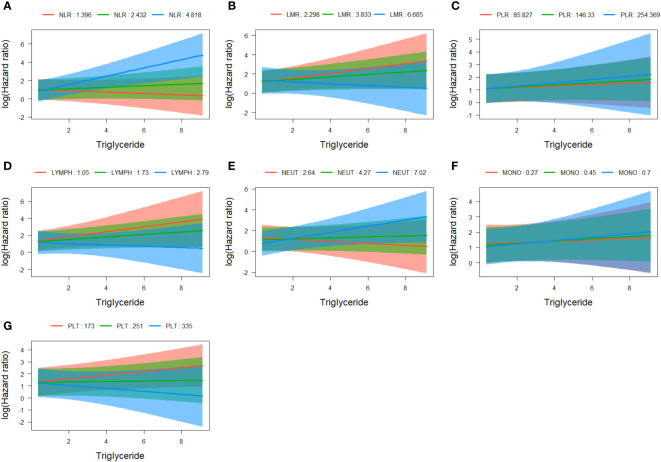
Assessment of interaction between triglyceride and NLR **(A)**, LMR **(B)**, PLR **(C)**, lymphocytes **(D)**, neutrophils **(E)**, monocytes **(F)**, and platelets **(G)**. NLR, neutrophil to lymphocyte ratio; LMR, lymphocyte to monocyte ratio; PLR, platelet to lymphocyte ratio.

Statistical interaction analyses of COX regression adjusted for age, sex, and overall stage revealed evidence of statistical interaction between triglyceride and NLR (HR=1.167, 95% CI=1.062-1.283, p=0.001) (**Figure 6A**), as well as platelets (HR=0.998, 95% CI=0.997-1.000, p=0.044) (**Figure 6G**). However, no significant interactions were observed between triglyceride and LMR, PLR, lymphocytes, neutrophils, and monocytes (**Figures 6B-F**; **Supplementary Table 4**).

**Figure 7:**
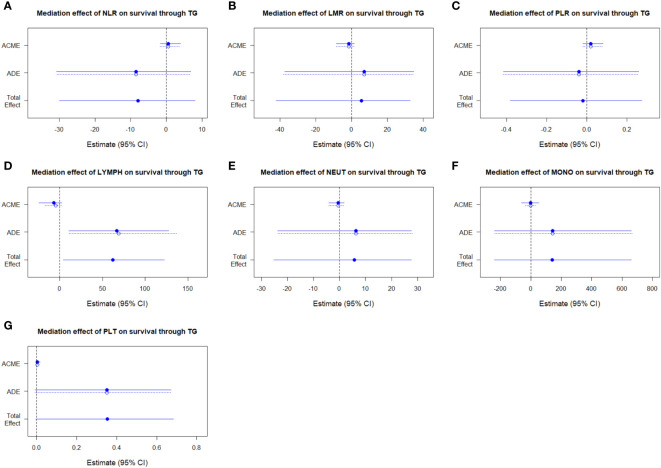
Assessment of mediation between triglyceride and NLR **(A)**, LMR **(B)**, PLR **(C)**, lymphocytes **(D)**, neutrophils **(E)**, monocytes **(F)**, and platelets **(G)**. NLR, neutrophil to lymphocyte ratio; LMR, lymphocyte to monocyte ratio; PLR, platelet to lymphocyte ratio; ACME, average causal mediation effects; ADE, average direct effects.

We established the regression models of survival outcome, observational variables (including age, sex, overall stage, and the parameters of peripheral blood cells), and mediator variable (triglyceride), and used the medicate function of R package “mediation” to estimate the average causal mediation effects (indirect effects) of NLR, LMR, PLR, lymphocytes, neutrophils, monocytes, or platelets on OS of NPC patients through the mediator triglyceride. The results indicated that triglyceride did not exhibit any medicating effects in the case of those inflammatory markers, respectively (**Figure 7**; **Supplementary Table 5**).

### 3.6 Computational analysis shows the association between intracellular triglyceride metabolism and immune cell infiltration

**Figure 8:**
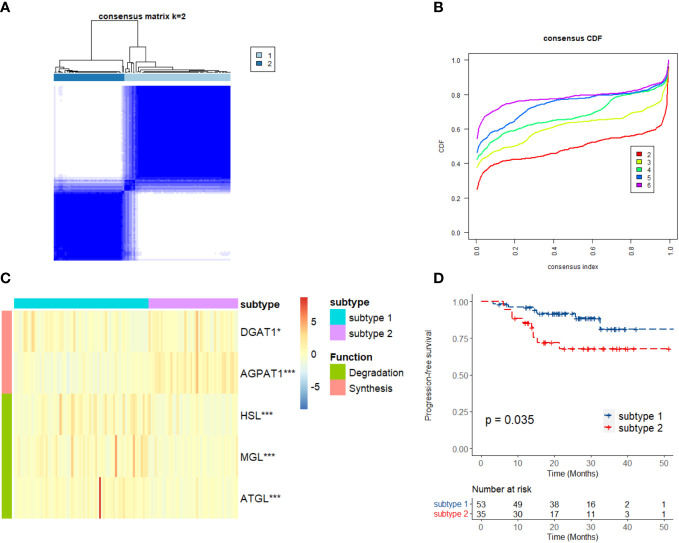
Consensus Clustering of triglyceride metabolism in GSE102349. **(A)** Consensus clustering matrix for k = 2. **(B)** Consensus clustering cumulative distribution function for k = 2–6. **(C)** Expression differences in the triglyceride metabolism genes between two subtypes. **(D)** Kaplan–Meier curves of progression-free survival for two subtypes in NPC. Subtype 1: low anabolism and high catabolism of triglyceride; Subtype 2: high anabolism and low catabolism of triglyceride. *p < 0.05, ***p < 0.001.

**Figure 9:**
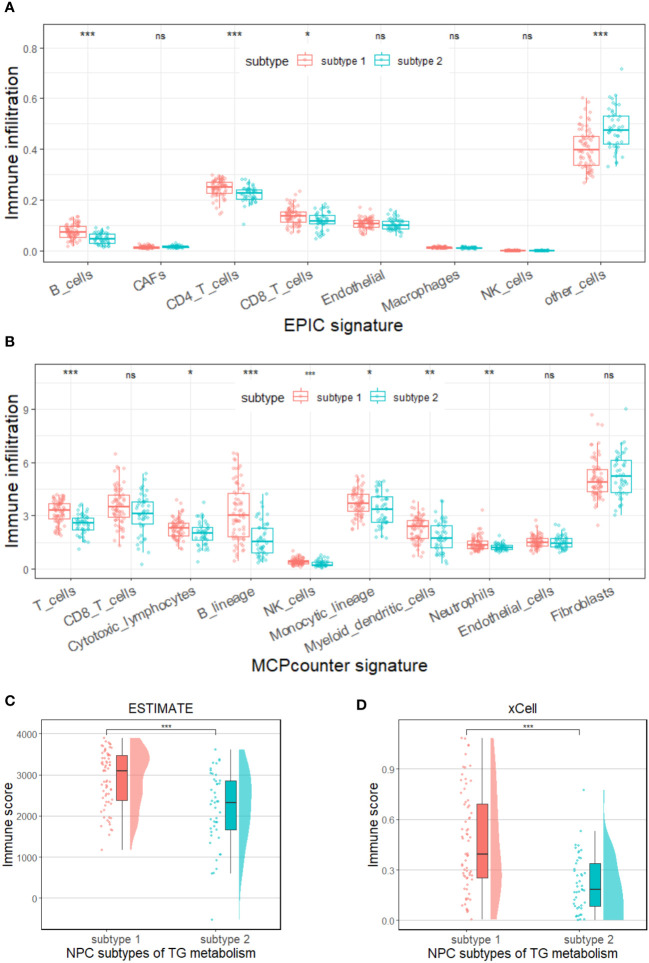
Immune status in two subtypes of triglyceride metabolism in the GSE102349 dataset of NPC. Differences in immune cell infiltration between the two subtypes were analyzed by using EPIC **(A)** or MCP-counter **(B)** signature. Differences in immune scores between the two subtypes were analyzed by using the ESTIMATE **(C)** and xCell **(D)** algorithms. Subtype 1: low anabolism and high catabolism of triglyceride; Subtype 2: high anabolism and low catabolism of triglyceride. TG: triglyceride, *p < 0.05, **p < 0.01, ***p < 0.001, ns, no statistical significance.

It is well known that the process of triglyceride metabolism is completed by a series of genes. To further explore the association between triglyceride metabolism and inflammatory markers (lymphocytes, neutrophils, and monocytes), computational analysis was conducted based on the GEO dataset GSE102349. We firstly performed RCS analysis and found a gradual increase in the disease progression risk of NPC patients with increasing expression of two key genes involved in the triglyceride anabolism (AGPAT1 and DGAT1), while a gradual decrease in the disease progression risk as observed with increasing expression of three key genes involved in the triglyceride catabolism (ATGL, HSL, and MGL) (**Supplementary Figure 5**). We then classified the NPC cases in GSE102349 into two subtypes according to the transcription level of the five triglyceride metabolism-related genes, via Consensus Clustering (**Figures 8A, B**). A statistical difference was found between the expressing patterns of the two subtypes. That is to say, NPC patients in subtype 1 showed a low triglyceride synthesis (lower expression of AGPAT1 and DGAT1) and high triglyceride degradation (higher expression of ATGL, HSL, and MGL) (p < 0.05), and reversely, NPC patients in subtype 2 showed a high triglyceride synthesis (higher expression of AGPAT1 and DGAT1) and low triglyceride degradation (lower expression of ATGL, HSL, and MGL) (p < 0.05) (**Figure 8C**). What’s more, NPC patients in subtype 2 had poorer PFS compared to those in subtype 1 (**Figure 8D**), consistent with our hospital-based survival study in NPC patients with high serum levels of triglyceride (**Figure 2A**). Enlightened by the interaction between triglyceride and NLR, we next tried to explore the association between NPC subtypes and immune cell infiltration. It was noted that NPC subtype 1 exhibited higher proportions of B cells and T cells, as well as a higher immune score (**Figures 9A-D**), and the proportions of monocytic lineage and neutrophils were significantly higher in NPC subtype 1 than in subtype 2 (**Figure 9B**), indicating a significant association between intracellular triglyceride metabolism and immune cell infiltration in NPC tissues. However, further validation in a larger sample size is needed and related mechanisms are worth exploring.

## 4 Discussion

Nowadays, interaction among metabolism, inflammation, and cancer microenvironment, is becoming one of the hot spots in cancer research. In the present study, we first examined the correlation between blood triglyceride, inflammatory markers, and the OS of NPC patients, and found that high levels of triglyceride, NLR, and PLR, as well as low LMR, predicted poor survival outcomes of NPC patients. A machine learning model, namely TI score, was then developed by integrating the circulating level of triglyceride, NLR, LMR, and PLR, and showed favorable prognostic function in NPC, superior to the other score systems such as CONUT score or PNI. Statistical interaction was found between triglyceride and NLR in the association with OS of NPC patients. Additionally, computational analysis showed that NPC patients with low triglyceride synthesis and high triglyceride degradation were associated with higher infiltration of B cells and T cells, and higher immune scores, proposing a potential interaction between triglyceride metabolism and NPC tumor microenvironment.

Metabolism dysregulation is a critical hallmark of cancer. Obesity and excessive consumption of high-fat diets can alter metabolism and cellular status in healthy tissues, increasing the susceptibility to cancer (31). What’s more, tumor cells remodulate lipid metabolism in order to grow quickly and progress aggressively (32). Triglyceride, as the main constituents of human body fat, enhances tumor cell proliferation and growth in the context of hypoxia and hypoperfusion (33). Higher level of serum triglycerides is associated with increased risks of breast, lung, rectal, and thyroid cancer (34, 35). Overactivation of triglyceride synthesis and downregulation of triglyceride degradation have been noted in the tumorigenesis of colorectal cancer (CRC) (36). Furthermore, high level of serum triglyceride is closely associated with poor prognosis in non-small cell lung cancer (37). Similar results are found in NPC. For instance, an Italian study reported that a high dietary intake of cholesterol could increase the risk of NPC (38). In our previous study, an epidemiological survey indicated that a higher intake of animal-based foods was associated with an elevated risk of NPC (39), and a hospital-based case-control study showed significantly different plasma lipidomic profiles between NPC patients and controls (5). Recent research also found that triglyceride was a potential risk factor for poor OS of non-metastatic NPC and eye metastases in male NPC patients (40, 41). In line with the above studies, the present study showed that high serum triglyceride was positively associated with the poor survival of NPC patients, which might be a prognostic predictor of NPC. Considering there is no available public dataset containing OS information on NPC cases, we tried to analyze the association between triglyceride metabolism and progression-free survival in NPC based on the GEO dataset GSE102349. As shown by bioinformatics analysis, high intracellular triglyceride synthesis (higher expression of AGPAT1 and DGAT1) and low triglyceride degradation (lower expression of ATGL, HSL, and MGL) were positively related to the disease progression of NPC. This was consistent with our previous study, in which obvious lipid droplet accumulation and its abilities to promote cell proliferation and invasion were found in NPC (42), and siRNA silencing of ATGL exhibited an increase of lipid droplets in NPC cells (43). We attempted to feed the C17 NPC patient-derived xenograft (PDX) model with a high-fat diet for 14 days and found that the high-fat diet accelerated NPC proliferation (data unpublished). To some extent, it was indicated that peripheral blood triglyceride levels may affect the progression of NPC.

Blood inflammatory markers have a strong prognostic value in many types of cancer. Given their easy accessibility and low costs in clinical practice, biomarkers such as NLR, RLR, and LMR, are widely evaluated for their prognostic function. It has been shown that the NLR and PLR can reliably predict the OS and PFS in patients with ovarian, prostate, or head and neck cancers (9, 11, 12). A low LMR is also independently associated with worse OS in CRC patients (44). There are also accumulated reports about their application in NPC. NPC patients with higher NLR, PLR, systemic immune index, and systemic inflammation response index, and lower LMR experienced poorer OS or PFS (14–16, 45). In this study, single-marker analysis revealed that NLR and PLR were associated with an increased risk of death, while LMR was associated with a decreased risk of death in NPC, further supporting the roles of NLR, PLR, and LMR in the prognosis of NPC.

Machine learning is widely applied in improving the accuracy of cancer susceptibility, recurrence, and survival prediction (46–48). As one of the popular machine learning algorithms, the tree-based RSF possessed a certain anti-noise ability due to the introduction of randomness. RSF models have been constructed for prognostic prediction of NPC, by applying microRNA, or tumor infiltrating lymphocytes (49, 50). Yet, to our knowledge, there is no RSF model based on lipid metabolism and inflammation. In this study, we generated a TI score by RSF, based on the circulating triglyceride, NLR, PLR, and LMR, which were identified through univariate analysis. The TI score worked excellently not only in survival prediction but also showed a significant positive association with tumor burden and distant metastasis in NPC, indicating its potential power in clinical application.

It is well-known that remodification of metabolism, inflammation, and immune microenvironment are critical biomarkers of cancer (4), and there is a complicated crosstalk among the three. Serum triglyceride levels have been associated with oxidative stress and chronic inflammation, which may play a potential role in the proliferation and progression of cancer cells (51). Additionally, cholesterol and fatty acids can regulate T cell differentiation through the activation of multiple signals (52). Low-grade inflammation induced by obesity, hyperglycemia, and excessive lipid accumulation is usually systemic and can promote the risk of many different cancers (53, 54). Interestingly, lipid-accumulating lung mesenchymal cells promote the metastasis of breast cancer cells by metabolic rewiring of cancer cells and natural killer cells, partially via the IL-1β-HILPDA-ATGL axis (55). Herein, our study indicated that there was an interaction between triglyceride and NLR, and NPC tissues with active triglyceride synthesis exhibited low immune cell infiltration, providing new clues for the mechanism of immune evasion in NPC.

Increasing evidence declare that lipids are associated with many immune cells and their phenotypic transformation, and tumor lipid metabolism is reprogrammed to promote immunosuppression, leading to immune escape (56). As the cancer-stromal interactions are intensified in the process of tumor progression, fatty acids secreted into the microenvironment would affect the function and phenotype of infiltrating immune cells (57). Studies showed that triglyceride-induced monocyte death by activating caspase-8/caspase-2/DNA damage pathways (58), and triglyceride-rich lipoprotein-induced monocyte-derived dendritic cells activation through ApoB48R upregulation in a fatty acid-dependent manner (59). Moreover, the environment enriched in lipid-dependent fatty acids might induce the phenotype of tumor-associated macrophages (TAMs), which have the characteristics of M2-like macrophages and exert multiple pro-tumoral properties (60). Abnormal accumulation of cholesterol in TAMs induces upregulation of ABCA1/G1 cholesterol efflux receptors and induces mitochondrial damage through oxidation product 7-ketocholesterol, which leads to TAMs phagocytic fragility and inhibits tumor necrosis factor signaling in TAMs (61). Lipid molecules such as triglyceride and cholesterol not only play a role in the metabolism and signal transduction of cancer cells but also participate in the response of immune cells recruited by tumors. Therefore, further elucidating the complex interactions between the different cell types and how lipids change their responses to each other will open up a road for NPC patients to improve their treatment and outcome.

There are some limits in this study. First, some patients were not included in this study since the blood lipid test before treatment was not necessary. However, the age and sex distribution of the enrolled patients did not deviate from the characteristics of the population in high-risk areas, so there might be only limited selective bias in this cohort. Second, the generalizability of the TI score was somewhat restricted, since the data were retrospectively collected from a single hospital and the model has not undergone external validation. It will be essential to carry out a large-scale multicenter study for validation in the future.

In the end, we developed a TI score via RSF, which was based on the convenience and cost-effectiveness of clinical parameters (triglyceride, NLR, RLR, and LMR), and generated a nomogram to accurately predict survival in NPC patients. What’s more, we will further investigate the influence of triglyceride metabolism on NPC development, partially due to the dysregulation of immune cell infiltration, which might thread light on the personalized prognosis and treatment strategies of NPC.

## Data availability statement

The raw data supporting the conclusions of this article will be made available by the authors, without undue reservation.

## Ethics statement

The studies involving humans were approved by the Ethics Committee of Wuzhou Red Cross Hospital. The studies were conducted in accordance with the local legislation and institutional requirements. The ethics committee/institutional review board waived the requirement of written informed consent for participation from the participants or the participants’ legal guardians/next of kin because The informed consent was waived due to its retrospective design.

## Author contributions

JL: Formal analysis, Writing – original draft, Conceptualization, Data curation. YY: Conceptualization, Data curation, Formal analysis, Writing – original draft. YC: Formal analysis, Writing – original draft, Funding acquisition, Investigation. HJ: Data curation, Writing – review & editing. WQ: Data curation, Writing – review & editing. YL: Data curation, Writing – review & editing. XZ: Funding acquisition, Writing – review & editing. ZZ: Funding acquisition, Writing – review & editing. XX: Conceptualization, Supervision, Validation, Writing – review & editing. BZ: Conceptualization, Supervision, Validation, Writing – review & editing.

## Supplementary material

Supplementary Figure 1 Restricted cubic spline analysis of the relation of triglyceride, cholesterol, HDL-C, LDL-C, lymphocytes, neutrophils, monocytes, platelets, NLR, LMR, and PLR with NPC patients’ mortality.

Supplementary Figure 2 Construction of random survival forest models based on triglyceride (TG), neutrophil to lymphocyte ratio (NLR), lymphocyte to monocyte ratio (LMR), and platelet to lymphocyte ratio (PLR) in the training set.

Supplementary Figure 3 Kaplan-Meier and time-dependent ROC curves analyses based on TI score of overall survival of NPC patients in the male subgroup **(A, C)** and female subgroup **(B, D)** of the entire set. TI: Triglyceride-inflammation.

Supplementary Figure 4 Kaplan-Meier and time-dependent ROC curves analyses based on CONUT score **(A, C)** or PNI **(B, D)** of overall survival of NPC patients in the albumin subset.

Supplementary Figure 5 Restricted cubic spline analysis of the relation of the mRNA expressions of the key genes involved in the anabolism (AGPAT1 and DGAT1) and catabolism (ATGL, HSL, and MGL) of triglyceride with disease progression of NPC in the GSE102349 dataset.

## References

[1] Chang ET Ye W Zeng YX Adami HO. The evolving epidemiology of nasopharyngeal carcinoma. Cancer Epidemiol Biomarkers Prev. (2021) 30:1035–47. doi: 10.1158/1055-9965.EPI-20-1702 33849968

[2] Chen YP Chan A Le QT Blanchard P Sun Y Ma J. Nasopharyngeal carcinoma. Lancet. (2019) 394:64–80. doi: 10.1016/S0140-6736(19)30956-0 31178151

[3] Ferlay J Ervik M Lam F Laversanne M Colombet M Mery L. Data from: Global cancer observatory: cancer today (version 1.1) (2024). Available online at: https://gco.iarc.fr/today.

[4] Hanahan D. Hallmarks of cancer: new dimensions. Cancer Discovery. (2022) 12:31–46. doi: 10.1158/2159-8290.CD-21-1059 35022204

[5] Huang Y Liang J Hu W Liang Y Xiao X Zhao W. Integration profiling between plasma lipidomics, epstein-barr virus and clinical phenomes in nasopharyngeal carcinoma patients. Front Microbiol. (2022) 13:919496. doi: 10.3389/fmicb.2022.919496 35847074 PMC9281874

[6] Tang Q Hu QY Piao YF Hua YH. Correlation between pretreatment serum LDL-cholesterol levels and prognosis in nasopharyngeal carcinoma patients. Onco Targets Ther. (2016) 9:2585–91. doi: 10.2147/OTT.S98079 PMC4860996 27217776

[7] Wang CT Chen MY Guo X Guo L Mo HY Qian CN. Association between pretreatment serum high-density lipoprotein cholesterol and treatment outcomes in patients with locoregionally advanced nasopharyngeal carcinoma treated with chemoradiotherapy: findings from a randomised trial. J Cancer. (2019) 10:3618–23. doi: 10.7150/jca.32621 PMC6636306 31333778

[8] Chen X Li YX Cao X Qiang MY Liang CX Ke LR. Widely targeted quantitative lipidomics and prognostic model reveal plasma lipid predictors for nasopharyngeal carcinoma. Lipids Health Dis. (2023) 22:81. doi: 10.1186/s12944-023-01830-2 37365637 PMC10294458

[9] Zhang CL Jiang XC Li Y Pan X Gao MQ Chen Y. Independent predictive value of blood inflammatory composite markers in ovarian cancer: recent clinical evidence and perspective focusing on NLR and PLR. J Ovarian Res. (2023) 16:36. doi: 10.1186/s13048-023-01116-2 36759864 PMC9912515

[10] Misiewicz A Dymicka-Piekarska V. Fashionable, but what is their real clinical usefulness? NLR, LMR, and PLR as a promising indicator in colorectal cancer prognosis: A systematic review. J Inflammation Res. (2023) 16:69–81. doi: 10.2147/JIR.S391932 PMC9833126 36643953

[11] Zhou M Liang J Hui J Xu J. Inflammation-related indicators have a potential to increase overall quality of the prostate cancer management: a narrative review. Transl Androl Urol. (2023) 12:809–22. doi: 10.21037/tau-23-55 PMC10251092 37305618

[12] Kumarasamy C Tiwary V Sunil K Suresh D Shetty S Muthukaliannan GK. Prognostic utility of platelet-lymphocyte ratio, neutrophil-lymphocyte ratio and monocyte-lymphocyte ratio in head and neck cancers: A detailed PRISMA compliant systematic review and meta-analysis. Cancers (Basel). (2021) 13:4166. doi: 10.3390/cancers13164166 34439320 PMC8393748

[13] Hu J Chen J Ou Z Chen H Liu Z Chen M. Neoadjuvant immunotherapy, chemotherapy, and combination therapy in muscle-invasive bladder cancer: A multi-center real-world retrospective study. Cell Rep Med. (2022) 3:100785. doi: 10.1016/j.xcrm.2022.100785 36265483 PMC9729796

[14] Lu A Li H Zheng Y Tang M Li J Wu H. Prognostic significance of neutrophil to lymphocyte ratio, lymphocyte to monocyte ratio, and platelet to lymphocyte ratio in patients with nasopharyngeal carcinoma. BioMed Res Int. (2017) 2017:3047802. doi: 10.1155/2017/3047802 28321405 PMC5340935

[15] Chen Y Sun J Hu D Zhang J Xu Y Feng H. Predictive value of pretreatment lymphocyte-to-monocyte ratio and platelet-to-lymphocyte ratio in the survival of nasopharyngeal carcinoma patients. Cancer Manag Res. (2021) 13:8767–79. doi: 10.2147/CMAR.S338394 PMC8633848 34866938

[16] Xu F Ni W Hua X Xu C Chen J Cao W. A single center retrospective study assessing the prognostic significance of pre-treatment neutrophil/lymphocyte ratio in locally advanced nasopharyngeal carcinoma. Transl Cancer Res. (2023) 12:1672–83. doi: 10.21037/tcr-23-528 PMC10425666 37588746

[17] Zhang L MacIsaac KD Zhou T Huang PY Xin C Dobson JR. Genomic analysis of nasopharyngeal carcinoma reveals TME-based subtypes. Mol Cancer Res. (2017) 15:1722–32. doi: 10.1158/1541-7786.MCR-17-0134 28851814

[18] Racle J de Jonge K Baumgaertner P Speiser DE Gfeller D. Simultaneous enumeration of cancer and immune cell types from bulk tumor gene expression data. Elife. (2017) 6:e26476. doi: 10.7554/eLife.26476 29130882 PMC5718706

[19] Becht E Giraldo NA Lacroix L Buttard B Elarouci N Petitprez F. Estimating the population abundance of tissue-infiltrating immune and stromal cell populations using gene expression. Genome Biol. (2016) 17:218. doi: 10.1186/s13059-016-1070-5 27765066 PMC5073889

[20] Yoshihara K Shahmoradgoli M Martinez E Vegesna R Kim H Torres-Garcia W. Inferring tumour purity and stromal and immune cell admixture from expression data. Nat Commun. (2013) 4:2612. doi: 10.1038/ncomms3612 24113773 PMC3826632

[21] Aran D Hu Z Butte AJ. xCell: digitally portraying the tissue cellular heterogeneity landscape. Genome Biol. (2017) 18:220. doi: 10.1186/s13059-017-1349-1 29141660 PMC5688663

[22] Ignacio DUJ Gonzalez-Madrono A de Villar NG Gonzalez P Gonzalez B Mancha A. CONUT: a tool for controlling nutritional status. First validation in a hospital population. Nutr Hosp. (2005) 20:38–45. 15762418

[23] Jiang YM Huang ST Pan XB Ma JL Zhu XD. The prognostic nutritional index represents a novel inflammation-nutrition-based prognostic factor for nasopharyngeal carcinoma. Front Nutr. (2023) 10:1036572. doi: 10.3389/fnut.2023.1036572 36875852 PMC9977787

[24] Ishwaran H Kogalur UB. Consistency of random survival forests. Stat Probab Lett. (2010) 80:1056–64. doi: 10.1016/j.spl.2010.02.020 PMC2889677 20582150

[25] Blanche P Dartigues JF Jacqmin-Gadda H. Estimating and comparing time-dependent areas under receiver operating characteristic curves for censored event times with competing risks. Stat Med. (2013) 32:5381–97. doi: 10.1002/sim.5958 24027076

[26] Simon N Friedman J Hastie T Tibshirani R. Regularization paths for Cox's proportional hazards model via coordinate descent. J Stat Softw. (2011) 39:1–13. doi: 10.18637/jss.v039.i05 PMC4824408 27065756

[27] Breheny P Burchett W. Visualization of regression models using visreg. R J. (2017) 9:56–71. doi: 10.32614/RJ-2017-046

[28] Tingley D Yamamoto T Hirose K Keele L Imai K. mediation: R package for causal mediation analysis. J Stat Softw. (2014) 59:1–38. doi: 10.18637/jss.v059.i05 26917999

[29] Wilkerson MD Hayes DN. ConsensusClusterPlus: a class discovery tool with confidence assessments and item tracking. Bioinformatics. (2010) 26:1572–73. doi: 10.1093/bioinformatics/btq170 PMC2881355 20427518

[30] Zeng D Ye Z Shen R Yu G Wu J Xiong Y. IOBR: multi-omics immuno-oncology biological research to decode tumor microenvironment and signatures. Front Immunol. (2021) 12:687975. doi: 10.3389/fimmu.2021.687975 34276676 PMC8283787

[31] Martin-Perez M Urdiroz-Urricelqui U Bigas C Benitah SA. The role of lipids in cancer progression and metastasis. Cell Metab. (2022) 34:1675–99. doi: 10.1016/j.cmet.2022.09.023 36261043

[32] Bian X Liu R Meng Y Xing D Xu D Lu Z. Lipid metabolism and cancer. J Exp Med. (2021) 218:e20201606. doi: 10.1084/jem.20201606 33601415 PMC7754673

[33] Snaebjornsson MT Janaki-Raman S Schulze A. Greasing the wheels of the cancer machine: the role of lipid metabolism in cancer. Cell Metab. (2020) 31:62–76. doi: 10.1016/j.cmet.2019.11.010 31813823

[34] Kapil U Bhadoria AS Sareen N Singh P Dwivedi SN. Total cholesterol and triglyceride levels in patients with breast cancer. J Breast Cancer. (2013) 16:129–30. doi: 10.4048/jbc.2013.16.1.129 PMC3625762 23593095

[35] Ulmer H Borena W Rapp K Klenk J Strasak A Diem G. Serum triglyceride concentrations and cancer risk in a large cohort study in Austria. Br J Cancer. (2009) 101:1202–06. doi: 10.1038/sj.bjc.6605264 PMC2768093 19690552

[36] Yarla N Madka V Rao C. Targeting triglyceride metabolism for colorectal cancer prevention and therapy. Curr Drug Targets. (2022) 23:628–35. doi: 10.2174/1389450122666210824150012 34431463

[37] Ma C Wang X Guo J Liu P. Prognostic significance of preoperative serum triglycerides and high-density lipoproteins cholesterol in patients with non-small cell lung cancer: a retrospective study. Lipids Health Dis. (2021) 20:69. doi: 10.1186/s12944-021-01492-y 34598703 PMC8487143

[38] Polesel J Negri E Serraino D Parpinel M Barzan L Libra M. Dietary intakes of carotenoids and other nutrients in the risk of nasopharyngeal carcinoma: a case-control study in Italy. Br J Cancer. (2012) 107:1580–83. doi: 10.1038/bjc.2012.413 PMC3493759 22968647

[39] Huang T Ploner A Chang ET Liu Q Cai Y Zhang Z. Dietary patterns and risk of nasopharyngeal carcinoma: a population-based case-control study in southern China. Am J Clin Nutr. (2021) 114:462–71. doi: 10.1093/ajcn/nqab114 PMC8326029 33963745

[40] Huang R Chen K Jiang Y Li L Zhu X. Development of prognostic nomogram based on lipid metabolic markers and lactate dehydrogenase in non-metastatic nasopharyngeal carcinoma. J Inflammation Res. (2023) 16:3093–107. doi: 10.2147/JIR.S416801 PMC10378618 37520664

[41] Xie Z Shao Y. The predictive value of serum lipids for eye metastases in male nasopharyngeal carcinoma patients. Biosci Rep. (2020) 40:R20201082. doi: 10.1042/BSR20201082 PMC7317591 32584390

[42] Zhou X Wei J Chen F Xiao X Huang T He Q. Epigenetic downregulation of the ISG15-conjugating enzyme UbcH8 impairs lipolysis and correlates with poor prognosis in nasopharyngeal carcinoma. Oncotarget. (2015) 6:41077–91. doi: 10.18632/oncotarget.6218 PMC4747391 26506425

[43] Zheng S Matskova L Zhou X Xiao X Huang G Zhang Z. Downregulation of adipose triglyceride lipase by EB viral-encoded LMP2A links lipid accumulation to increased migration in nasopharyngeal carcinoma. Mol Oncol. (2020) 14:3234–52. doi: 10.1002/1878-0261.12824 PMC7718958 33064888

[44] Yamamoto T Kawada K Obama K. Inflammation-related biomarkers for the prediction of prognosis in colorectal cancer patients. Int J Mol Sci. (2021) 22:8002. doi: 10.3390/ijms22158002 34360768 PMC8348168

[45] Li Q Yu L Yang P Hu Q. Prognostic value of inflammatory markers in nasopharyngeal carcinoma patients in the intensity-modulated radiotherapy era. Cancer Manag Res. (2021) 13:6799–810. doi: 10.2147/CMAR.S311094 PMC8418375 34512020

[46] Wang Y Deng Y Tan Y Zhou M Jiang Y Liu B. A comparison of random survival forest and Cox regression for prediction of mortality in patients with hemorrhagic stroke. BMC Med Inform Decis Mak. (2023) 23:215. doi: 10.1186/s12911-023-02293-2 37833724 PMC10576378

[47] Lin J Yin M Liu L Gao J Yu C Liu X. The development of a prediction model based on random survival forest for the postoperative prognosis of pancreatic cancer: A SEER-based study. Cancers (Basel). (2022) 14:4667. doi: 10.3390/cancers14194667 36230593 PMC9563591

[48] Xiao Z Song Q Wei Y Fu Y Huang D Huang C. Use of survival support vector machine combined with random survival forest to predict the survival of nasopharyngeal carcinoma patients. Transl Cancer Res. (2023) 12:3581–90. doi: 10.21037/tcr-23-316 PMC10774032 38192980

[49] Wibawa MS Zhou JY Wang R Huang YY Zhan Z Chen X. AI-based risk score from tumour-infiltrating lymphocyte predicts locoregional-free survival in nasopharyngeal carcinoma. Cancers (Basel). (2023) 15:5789. doi: 10.3390/cancers15245789 38136336 PMC10742296

[50] Zhang SQ Liu J Chen HB Dai WJ Zhou LQ Xie CW. A novel three-microRNA signature for predicting survival in patients with nasopharyngeal carcinoma. J Dent Sci. (2022) 17:377–88. doi: 10.1016/j.jds.2021.08.017 PMC8740092 35028061

[51] Neshat S Rezaei A Farid A Sarallah R Javanshir S Ahmadian S. The tangled web of dyslipidemia and cancer: Is there any association? J Res Med Sci. (2022) 27:93. doi: 10.4103/jrms.jrms_267_22 36685020 PMC9854911

[52] Cai F Jin S Chen G. The effect of lipid metabolism on CD4(+) T cells. Mediators Inflammation. (2021) 2021:6634532. doi: 10.1155/2021/6634532 PMC7806377 33505215

[53] Quail DF Olson OC Bhardwaj P Walsh LA Akkari L Quick ML. Obesity alters the lung myeloid cell landscape to enhance breast cancer metastasis through IL5 and GM-CSF. Nat Cell Biol. (2017) 19:974–87. doi: 10.1038/ncb3578 PMC6759922 28737771

[54] Quail DF Dannenberg AJ. The obese adipose tissue microenvironment in cancer development and progression. Nat Rev Endocrinol. (2019) 15:139–54. doi: 10.1038/s41574-018-0126-x PMC6374176 30459447

[55] Gong Z Li Q Shi J Liu ET Shultz LD Ren G. Lipid-laden lung mesenchymal cells foster breast cancer metastasis via metabolic reprogramming of tumor cells and natural killer cells. Cell Metab. (2022) 34:1960–76. doi: 10.1016/j.cmet.2022.11.003 PMC9819197 36476935

[56] Wu Y Pu X Wang X Xu M. Reprogramming of lipid metabolism in the tumor microenvironment: a strategy for tumor immunotherapy. Lipids Health Dis. (2024) 23:35. doi: 10.1186/s12944-024-02024-0 38302980 PMC10832245

[57] Corn KC Windham MA Rafat M. Lipids in the tumor microenvironment: From cancer progression to treatment. Prog Lipid Res. (2020) 80:101055. doi: 10.1016/j.plipres.2020.101055 32791170 PMC7674189

[58] Jung BC Kim HK Kim SH Kim YS. Triglyceride induces DNA damage leading to monocyte death by activating caspase-2 and caspase-8. Bmb Rep. (2023) 56:166–71. doi: 10.5483/BMBRep.2022-0201 PMC10068338 36593108

[59] Vazquez-Madrigal C Lopez S Grao-Cruces E Millan-Linares MC Rodriguez-Martin NM Martin ME. Dietary fatty acids in postprandial triglyceride-rich lipoproteins modulate human monocyte-derived dendritic cell maturation and activation. Nutrients. (2020) 12:3139. doi: 10.3390/nu12103139 33066622 PMC7656296

[60] Wu H Han Y Rodriguez SY Deng H Siddiqui S Treese C. Lipid droplet-dependent fatty acid metabolism controls the immune suppressive phenotype of tumor-associated macrophages. EMBO Mol Med. (2019) 11:e10698. doi: 10.15252/emmm.201910698 31602788 PMC6835560

[61] Wang S Yan W Kong L Zuo S Wu J Zhu C. Oncolytic viruses engineered to enforce cholesterol efflux restore tumor-associated macrophage phagocytosis and anti-tumor immunity in glioblastoma. Nat Commun. (2023) 14:4367. doi: 10.1038/s41467-023-39683-z 37474548 PMC10359270

