# Supplementary material for: Triglyceride-inflammation score established on account of random survival forest for predicting survival in patients with nasopharyngeal carcinoma: a retrospective study
Source: Front Immunol. 2024 Apr 26;15:1375931. doi: 10.3389/fimmu.2024.1375931 (PMC11082337; doi:10.3389/fimmu.2024.1375931)
Supplement: Supplementary file 1 [file Table_1.docx]

**Supplementary Table 1** Comparison between the Chinese 2008 staging system and the 8^th^ edition of UICC/AJCC TNM classification for NPC

| Classification | Chinese 2008 staging system | 8^th^ edition of UICC/AJCC TNM classification |
| --- | --- | --- |
| Primary tumor (T) |  |  |
| TX | -- | Primary tumor cannot be assessed |
| T0 | -- | No tumor identified, but EBV-positive cervical node(s) involvement |
| T1 | Tumor confined to nasopharynx | Nasopharynx, oropharynx or nasal cavity without parapharyngeal extension |
| T2 | Nasal cavity, oropharynx, parapharyngeal; parapharyngeal extension | Parapharyngeal extension, adjacent soft tissue involvement (medial pterygoid, lateral pterygoid, prevertebral muscles) |
| T3 | Skull base, medial pterygoid muscle extension | Bony structures (skull base, cervical vertebra) and/or paranasal sinuses |
| T4 | Cranial nerve, paranasal sinus, masticatory space excluding medial pterygoid muscle, intracranial (cavernous, dural meninges) extension | Intracranial extension, cranial nerve, hypopharynx, orbit, extensive soft tissue involvement (beyond the lateral surface of the lateral pterygoid muscle), parotid gland |
| Regional lymph nodes (N) |  |  |
| NX | -- | Regional lymph nodes cannot be assessed |
| N0 | No regional lymph node metastasis | No regional lymph node metastasis |
| N1 | N1a RLN involvement; N1b unilateral level Ib, II, III, and Va involvement and the maximum diameter <3 cm | Unilateral cervical, unilateral or bilateral retropharyngeal lymph nodes, above the caudal border of cricoid cartilage;≤6 cm |
| N2 | Bilateral level Ib, II, III, and Va or the maximum diameter >3 cm or with extranodal neoplastic spread | Bilateral metastasis in lymph node(s), 6 cm or less in greatest dimension, above the caudal border of cricoid cartilage |
| N3 | Level IV, Vb involvement | >6 cm and/or below caudal border of cricoid cartilage (regardless of laterality) |
| Distant metastasis (M) |  |  |
| M0 | No distant metastasis | No distant metastasis |
| M1 | Distant metastasis | Distant metastasis |
| Stage group |  |  |
| I | T1 N0 M0 | T1 N0 M0 |
| II | T1 N1a-1b M0, T2 N0-1b M0 | T2 N0-1 M0, T0-1 N1 M0 |
| III | T1-2 N2 M0, T3 N0-2 M0 | T3 N0-2 M0, T0-2 N2 M0 |
| IVA | T4 N0-2 M0, T1-4 N3 M0 | T4 or N3 M0 |
| IVB | Any T, any N M1 | Any T, any N M1 |

UICC: Union for International Cancer Control; AJCC: American Joint Committee on Cancer.

**Supplementary Table 2** Nutritional status assessment according to the controlling nutritional status (CONUT) scoring system

| Parameters | Malnutrition status | | | |
| --- | --- | --- | --- | --- |
|  | Normal | Light | Moderate | Severe |
| Serum albumin (g/dL) | ≥3.50 | 3.00–3.49 | 2.50–2.99 | <2.50 |
| Score | 0 | 2 | 4 | 6 |
| Total lymphocyte count (/mm^3^) | ≥1,600 | 1,200–1,599 | 800–1,199 | <800 |
| Score | 0 | 1 | 2 | 3 |
| Total cholesterol (mg/dL) | ≥180 | 140–179 | 100–139 | <100 |
| Score | 0 | 1 | 2 | 3 |
| Total score | 0–1 | 2–4 | 5–8 | 9–12 |

**Supplementary Table 3** Comparison of baseline characteristics between the entire set and the albumin subset

| Characteristics | Entire set (n=259) | Albumin subset (n=229) | p value |
| --- | --- | --- | --- |
| Age | 52.0 (44.0, 59.5) | 52.0 (44.0, 59.0) | 0.971 |
| Sex |  |  | 0.57 |
| Female | 68 (26.3%) | 55 (24.0%) |  |
| Male | 191 (73.7%) | 174 (76.0%) |  |
| T stage |  |  | 0.916 |
| T1 | 37 (15.1%) | 31 (14.1%) |  |
| T2 | 63 (25.7%) | 60 (27.3%) |  |
| T3 | 69 (28.2%) | 57 (25.9%) |  |
| T4 | 76 (31.0%) | 72 (32.7%) |  |
| Missing | 14 | 9 |  |
| N stage |  |  | 0.982 |
| N0 | 18 (7.3%) | 17 (7.7%) |  |
| N1 | 40 (16.3%) | 33 (15.0%) |  |
| N2 | 149 (60.8%) | 136 (61.8%) |  |
| N3 | 38 (15.5%) | 34 (15.5%) |  |
| Missing | 14 | 9 |  |
| M stage |  |  | 0.936 |
| M0 | 229 (93.5%) | 207 (94.1%) |  |
| M1 | 13 (5.3%) | 11 (5.0%) |  |
| Mx | 3 (1.2%) | 2 (0.9%) |  |
| Missing | 14 | 9 |  |
| Overall stage |  |  | 0.999 |
| I | 8 (3.3%) | 7 (3.2%) |  |
| II | 18 (7.3%) | 16 (7.3%) |  |
| III | 114 (46.5%) | 101 (45.9%) |  |
| IV | 105 (42.9%) | 96 (43.6%) |  |
| Missing | 14 | 9 |  |
| Status |  |  | 0.356 |
| Alive | 165 (63.7%) | 155 (67.7%) |  |
| Dead | 94 (36.3%) | 74 (32.3%) |  |
| Triglyceride (mmol/L) | 1.24 (0.93, 1.84) | 1.26 (0.94, 1.91) | 0.865 |
| Female | 1.00 (0.80, 1.38) | 0.98 (0.80, 1.45) | 0.984 |
| Male | 1.30 (0.97, 2.07) | 1.32 (0.97, 2.07) | 0.986 |
| CHOL (mmol/L) | 5.07 (4.25, 5.67) | 5.06 (4.19, 5.62) | 0.696 |
| Female | 4.59 (3.96, 5.56) | 4.57 (3.94, 5.39) | 0.718 |
| Male | 5.11 (4.40, 5.70) | 5.11 (4.37, 5.68) | 0.728 |
| HDL-C (mmol/L) | 1.22 (1.05, 1.45) | 1.21 (1.05, 1.45) | 0.745 |
| Female | 1.33 (1.18, 1.50) | 1.34 (1.17, 1.52) | 0.959 |
| Male | 1.19 (1.00, 1.39) | 1.18 (1.00, 1.39) | 0.813 |
| LDL-C (mmol/L) | 3.31 (2.59, 3.84) | 3.28 (2.57, 3.83) | 0.792 |
| Female | 2.94 (2.20, 3.74) | 2.96 (2.28, 3.64) | 0.881 |
| Male | 3.40 (2.73, 3.95) | 3.34 (2.68, 3.88) | 0.736 |
| WBC (10^9^/L) | 6.91 (5.60, 8.52) | 7.00 (5.70, 8.40) | 0.995 |
| Female | 6.75 (4.87, 8.00) | 5.80 (4.80, 7.80) | 0.41 |
| Male | 7.00 (5.92, 8.75) | 7.07 (6.10, 8.70) | 0.688 |
| NLR | 2.48 (1.83, 3.42) | 2.36 (1.75, 3.14) | 0.238 |
| Female | 2.23 (1.72, 3.57) | 2.16 (1.61, 3.01) | 0.238 |
| Male | 2.51 (1.88, 3.41) | 2.47 (1.83, 3.18) | 0.493 |
| LMR | 3.83 (2.98, 5.12) | 3.87 (3.09, 5.22) | 0.423 |
| Female | 4.29 (3.27, 5.89) | 4.45 (3.71, 6.29) | 0.326 |
| Male | 3.70 (2.95, 4.88) | 3.73 (2.98, 5.02) | 0.701 |
| PLR | 148 (109, 188) | 143 (108, 181) | 0.373 |
| Female | 160 (140, 223) | 157 (138, 201) | 0.523 |
| Male | 136 (107, 182) | 134 (106, 175) | 0.578 |

**Supplementary Table 4** Interaction between triglyceride and the inflammatory markers in the association with overall survival of NPC

| Interaction | Hazard ratio | Lower 95% CI | Upper 95% CI | P value |
| --- | --- | --- | --- | --- |
| TG*NLR | 1.167 | 1.062 | 1.283 | 0.001 |
| TG*LMR | 0.924 | 0.812 | 1.052 | 0.234 |
| TG*PLR | 1.000 | 0.998 | 1.003 | 0.757 |
| TG*Lymphocytes | 0.810 | 0.552 | 1.190 | 0.283 |
| TG*Neutrophils | 1.094 | 0.982 | 1.218 | 0.102 |
| TG*Monocytes | 1.163 | 0.387 | 3.496 | 0.788 |
| TG*Platelets | 0.998 | 0.997 | 1.000 | 0.044 |

TG, triglyceride; CI, confidence interval; NLR: neutrophil to lymphocyte ratio; LMR: lymphocyte to monocyte ratio; PLR: platelet to lymphocyte ratio.

**Supplementary Table 5** Mediation between triglyceride and the inflammatory markers in the association with overall survival of NPC

| Mediation | Estimated value | Lower 95% CI | Upper 95% CI | P value |
| --- | --- | --- | --- | --- |
| TG: NLR | 0.567 | -1.736 | 3.940 | 0.640 |
| TG: LMR | -1.523 | -8.724 | 1.510 | 0.410 |
| TG: PLR | 0.019 | -0.021 | 0.080 | 0.380 |
| TG: Lymphocytes | -5.616 | -20.522 | 2.230 | 0.170 |
| TG: Neutrophils | -0.524 | -4.161 | 1.750 | 0.730 |
| TG: Monocytes | -1.770 | -4.500 | 39.680 | 0.890 |
| TG: Platelets | 0.003 | -0.006 | 0.020 | 0.512 |

TG, triglyceride; CI, confidence interval; NLR: neutrophil to lymphocyte ratio; LMR: lymphocyte to monocyte ratio; PLR: platelet to lymphocyte ratio.
