# Supplementary figures and images for: Triglyceride-inflammation score established on account of random survival forest for predicting survival in patients with nasopharyngeal carcinoma: a retrospective study
Source: Front Immunol. 2024 Apr 26;15:1375931. doi: 10.3389/fimmu.2024.1375931 (PMC11082337; doi:10.3389/fimmu.2024.1375931)

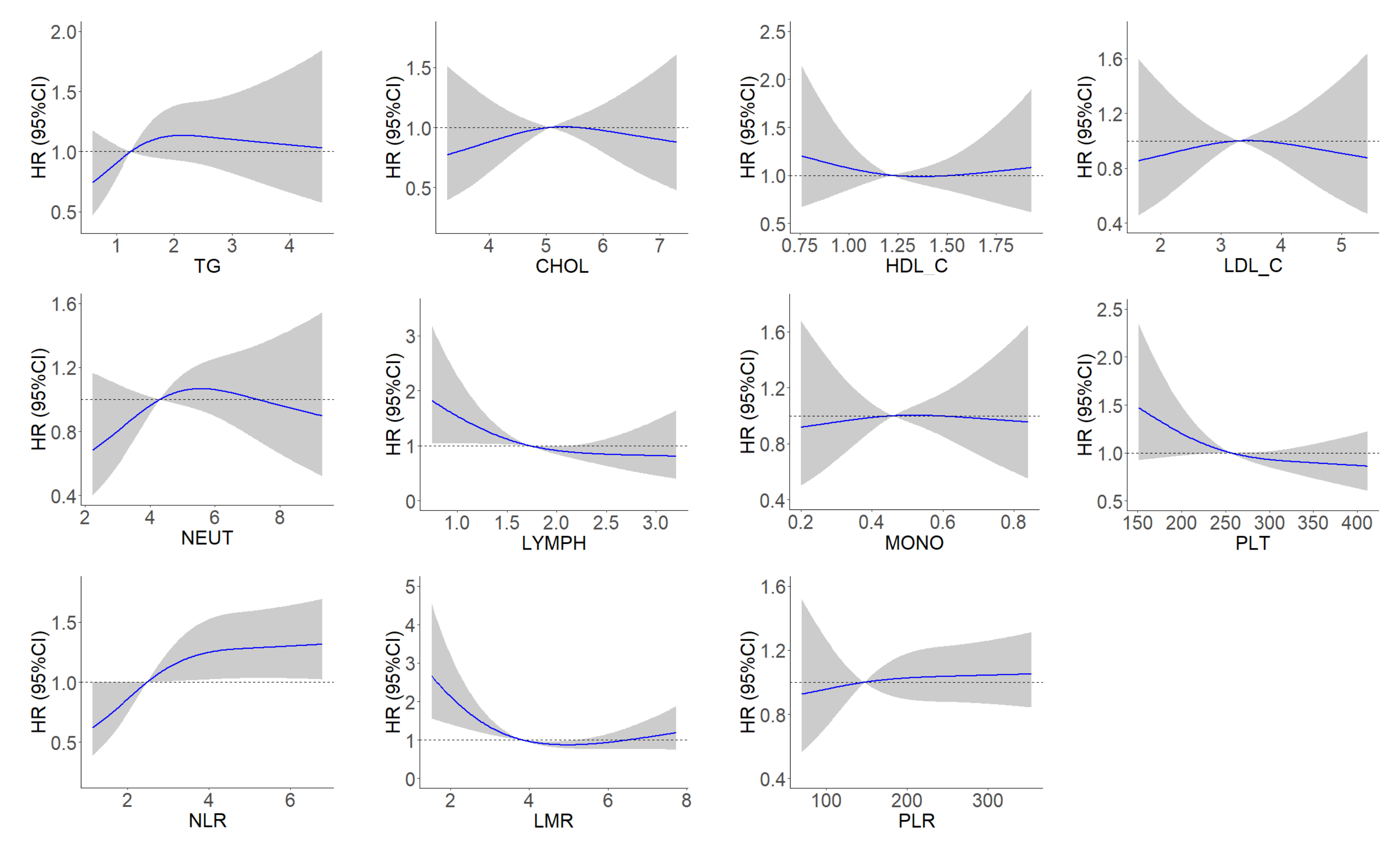

Supplement: Supplementary Figure 1 — Restricted cubic spline analysis of the relation of triglyceride, cholesterol, HDL-C, LDL-C, lymphocytes, neutrophils, monocytes, platelets, NLR, LMR, and PLR with NPC patients’ mortality. [file Image_1.tif]

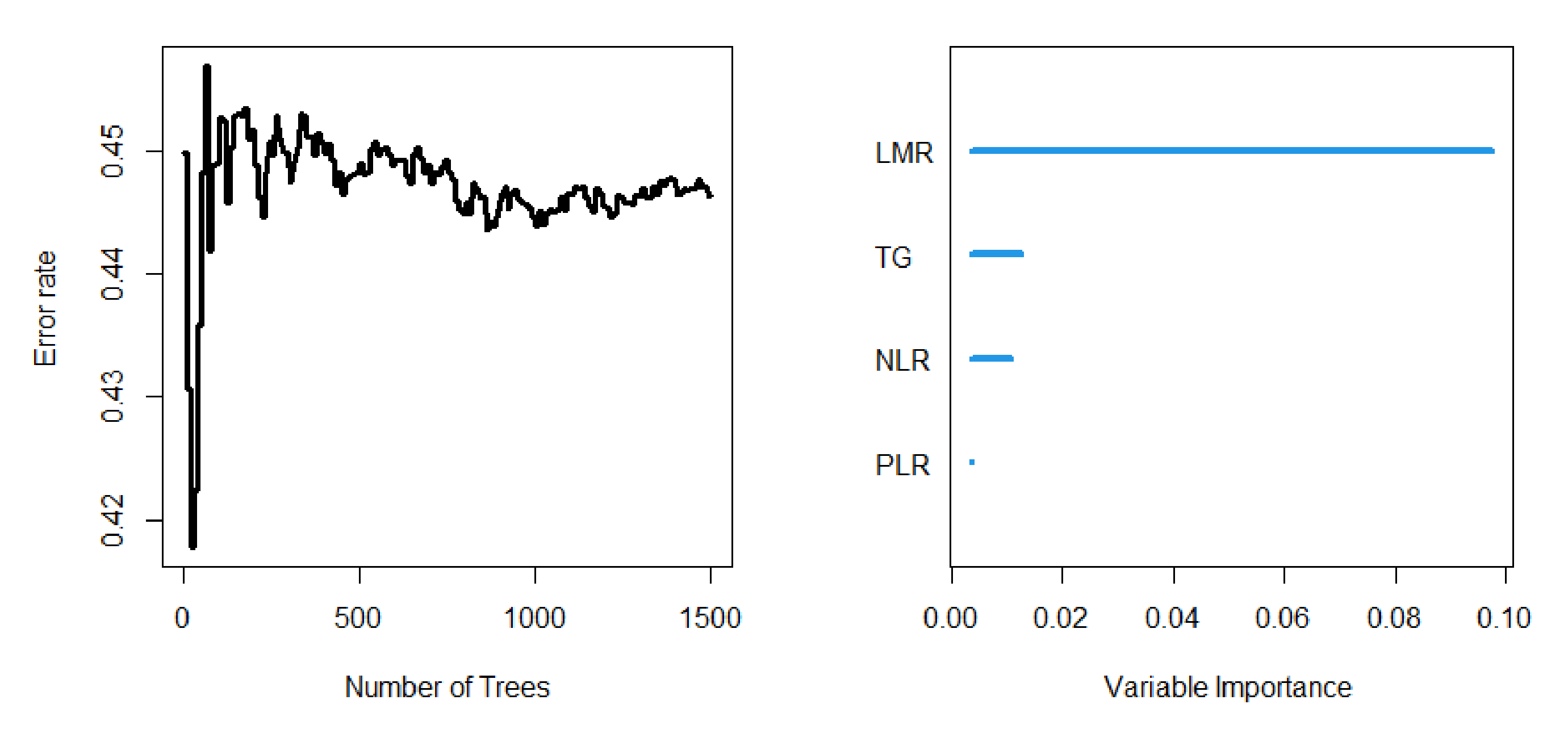

Supplement: Supplementary Figure 2 — Construction of random survival forest models based on triglyceride (TG), neutrophil to lymphocyte ratio (NLR), lymphocyte to monocyte ratio (LMR), and platelet to lymphocyte ratio (PLR) in the training set. [file Image_2.tif]

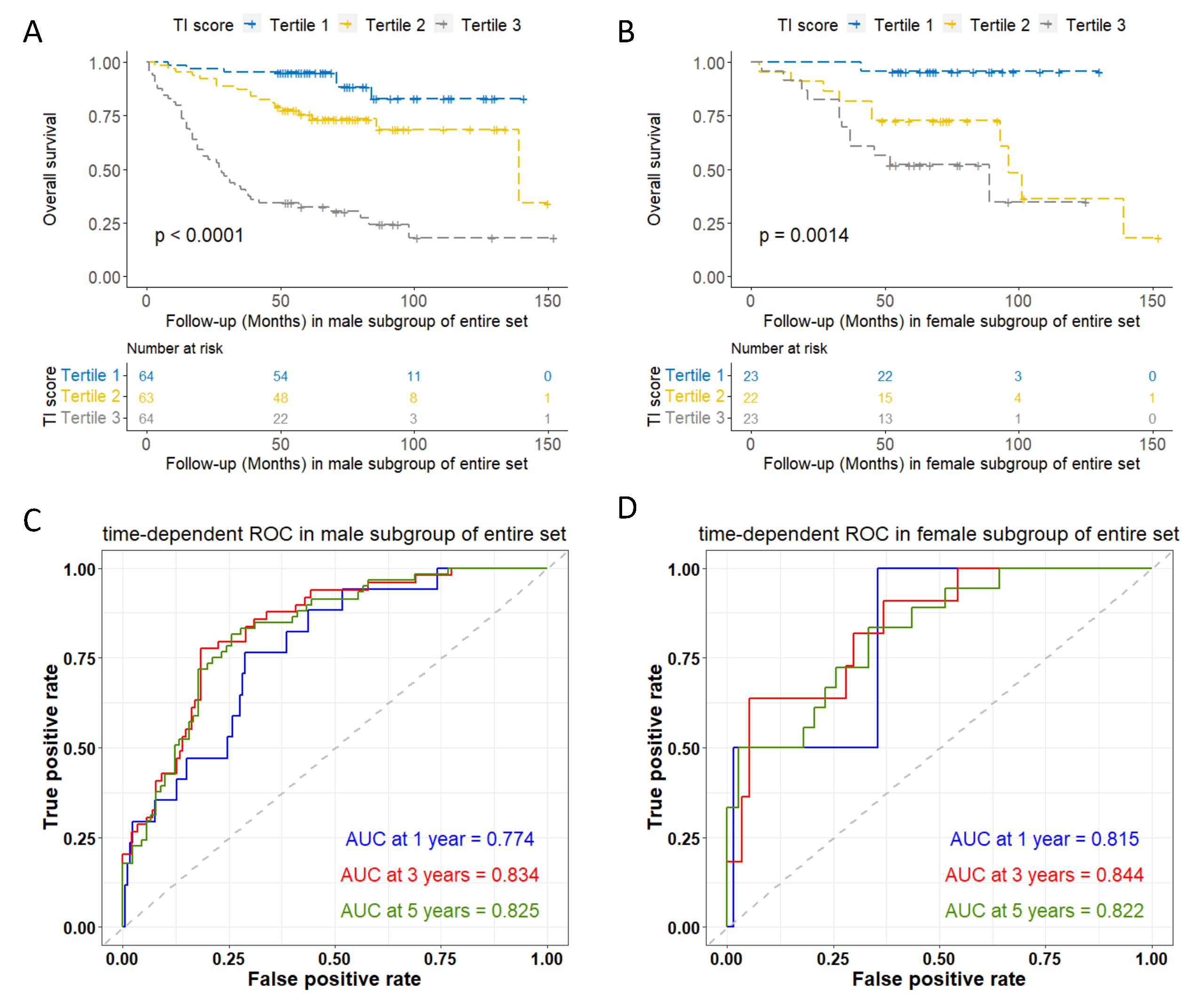

Supplement: Supplementary Figure 3 — Kaplan-Meier and time-dependent ROC curves analyses based on TI score of overall survival of NPC patients in the male subgroup (A, C) and female subgroup (B, D) of the entire set. TI: Triglyceride-inflammation. [file Image_3.tif]

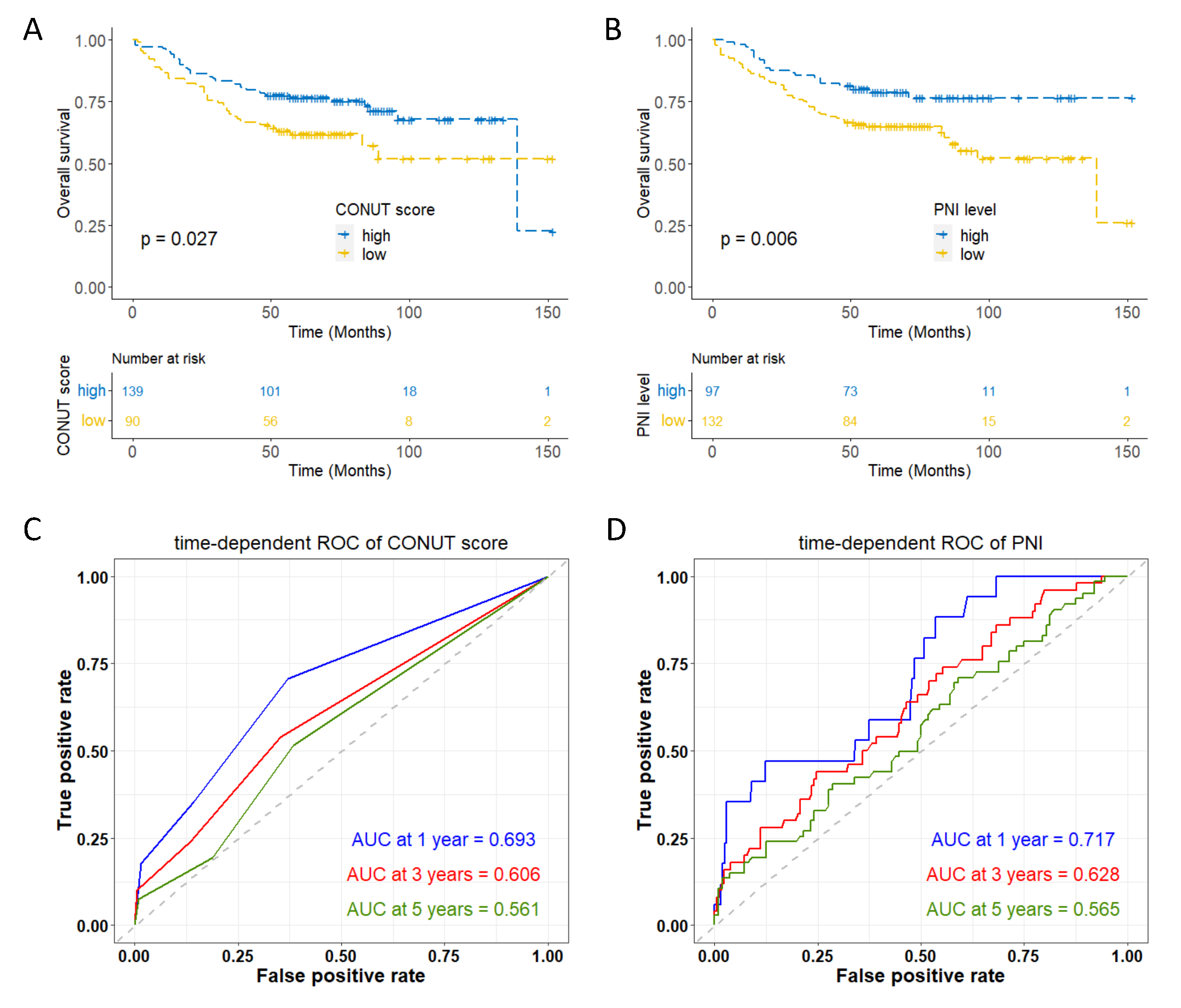

Supplement: Supplementary Figure 4 — Kaplan-Meier and time-dependent ROC curves analyses based on CONUT score (A, C) or PNI (B, D) of overall survival of NPC patients in the albumin subset. [file Image_4.tif]

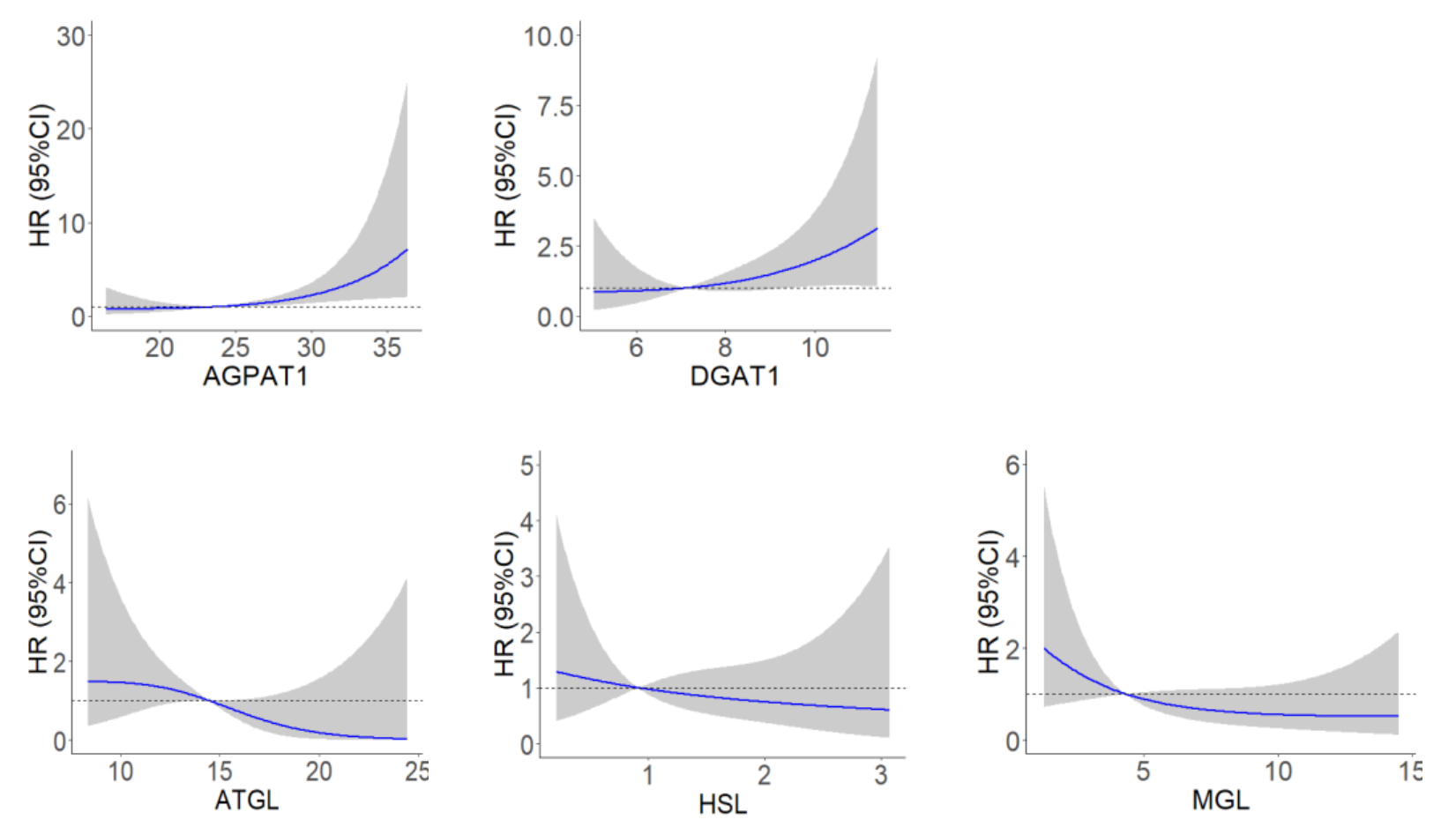

Supplement: Supplementary Figure 5 — Restricted cubic spline analysis of the relation of the mRNA expressions of the key genes involved in the anabolism (AGPAT1 and DGAT1) and catabolism (ATGL, HSL, and MGL) of triglyceride with disease progression of NPC in the GSE102349 dataset. [file Image_5.tif]
